# Could We Address the Interplay Between CD133, Wnt/β-Catenin, and TERT Signaling Pathways as a Potential Target for Glioblastoma Therapy?

**DOI:** 10.3389/fonc.2021.642719

**Published:** 2021-04-01

**Authors:** Amir Barzegar Behrooz, Amir Syahir

**Affiliations:** ^1^ Department of Biochemistry, Faculty of Biotechnology and Biomolecular Science, Universiti Putra Malaysia, Serdang, Malaysia; ^2^ MAKNA Cancer Research Laboratory, Institute of Bioscience, Universiti Putra Malaysia, Serdang, Malaysia

**Keywords:** glioblastoma stem cells, CD133, Wnt/β-catenin, PI3K/AKT/mTOR, telomerase

## Abstract

Glioblastoma multiforme (GBM) is one of the most lethal forms of primary brain tumors. Glioblastoma stem cells (GSCs) play an undeniable role in tumor development by activating multiple signaling pathways such as Wnt/β-catenin and PI3K/AKT/mTOR that facilitate brain tumor formation. CD133, a transmembrane glycoprotein, has been used to classify cancer stem cells (CSCs) in GBM. The therapeutic value of CD133 is a biomarker of the CSC in multiple cancers. It also leads to growth and recurrence of the tumor. More recent findings have confirmed the association of telomerase/TERT with Wnt/β-catenin and the PI3K/AKT/mTOR signaling pathways. Advance studies have shown that crosstalk between CD133, Wnt/β-catenin, and telomerase/TERT can facilitate GBM stemness and lead to therapeutic resistance. Mechanistic insight into signaling mechanisms downstream of surface biomarkers has been revolutionized by facilitating targeting of tumor-specific molecular deregulation. This review also addresses the importance of interplay between CD133, Wnt/β-catenin and TERT signaling pathways in GSCs and outlines the future therapeutic goals for glioblastoma treatment.

## Background

Glioblastoma multiform (GBM) is one of the calamitous kinds of aggressive primary glial brain tumor in adults, with a median overall survival between 10 to 20 months. Intriguingly, the recent study has demonstrated that outer radial glia-like cancer stem cells could impart to heterogenicity of GBM (1). The striking cellular-heterogeneity is one of the prominent hallmarks of GBM, and glioblastoma stem cells (GSCs) are placed at the apex of it (2). GSCs have been indicated to be involved in imperative processes of tumor growth, disseminated-metastases, chemo- and radio-therapy resistance and GBM relapse (3).

GSCs have been identified by many biomarkers of CSCs. CD133, also known as prominin-1, has been used as an essential marker for the detection of GSCs. Additionally, CD133 has been shown to be associated with GBM development, recurrence, and poor overall survival (4). Emerging observations have suggested that CD133 could act as a novel receptor for PI3K/AKT/mTOR pathway (5). Further, CD133-Wnt/β-catenin axis was gained attention in GBM as a stemness regulatory pathway and lead to resistance to chemo- and radio- therapies (6). Accumulating data have indicated that AKT might activate Wnt/β-catenin pathway (7, 8). Furthermore, the results of studies have confirmed the interaction of telomerase/TERT with PI3K/AKT/mTOR and Wnt/β-catenin in various cancers which predominately participating in tumor invasion and metastasis and epithelial to mesenchymal transition (EMT) (9).

Each component of the CD133, Wnt/β-catenin, and TERT signaling pathways play a crucial role in the normal brain. CD133 is present in epithelial cells throughout the body, including the mammary gland, testis, digestive tract, trachea, and placenta, and is expressed in stem cells. CD133 is also present in non-epithelial cells, such as rod photoreceptor cells, and in many cancers (10). The Wnt/β-catenin signaling has a pivotal role in neural development. The underlying neural scaffolding that makes the diverse cognitive functions of the cerebral cortex is generated by a set of basic developmental processes. Production and differentiation of the immense diversity of nervous system cells entail a range of main activities, ranging from regional progenitor specification, separation and expansion of neural precursor populations, neuron generation, movement of young neurons to suitable locations, the evolution of neuronal processes, and the forming of complex synaptic connections (11). For both physiological processes and the transformation of human cells by stabilizing telomere length, TERT activation is necessary. TERT’s telomere lengthening-independent roles contribute significantly to both physiological processes and cancer initiation or progression, including its effects on mitochondrial, ubiquitin-proteasomal (UPS) structures, gene transcription, expression of microRNA (miRNA), repair of DNA damage, and operation of RNA-dependent RNA polymerase (RdRP) (12).

**Figure 1:**
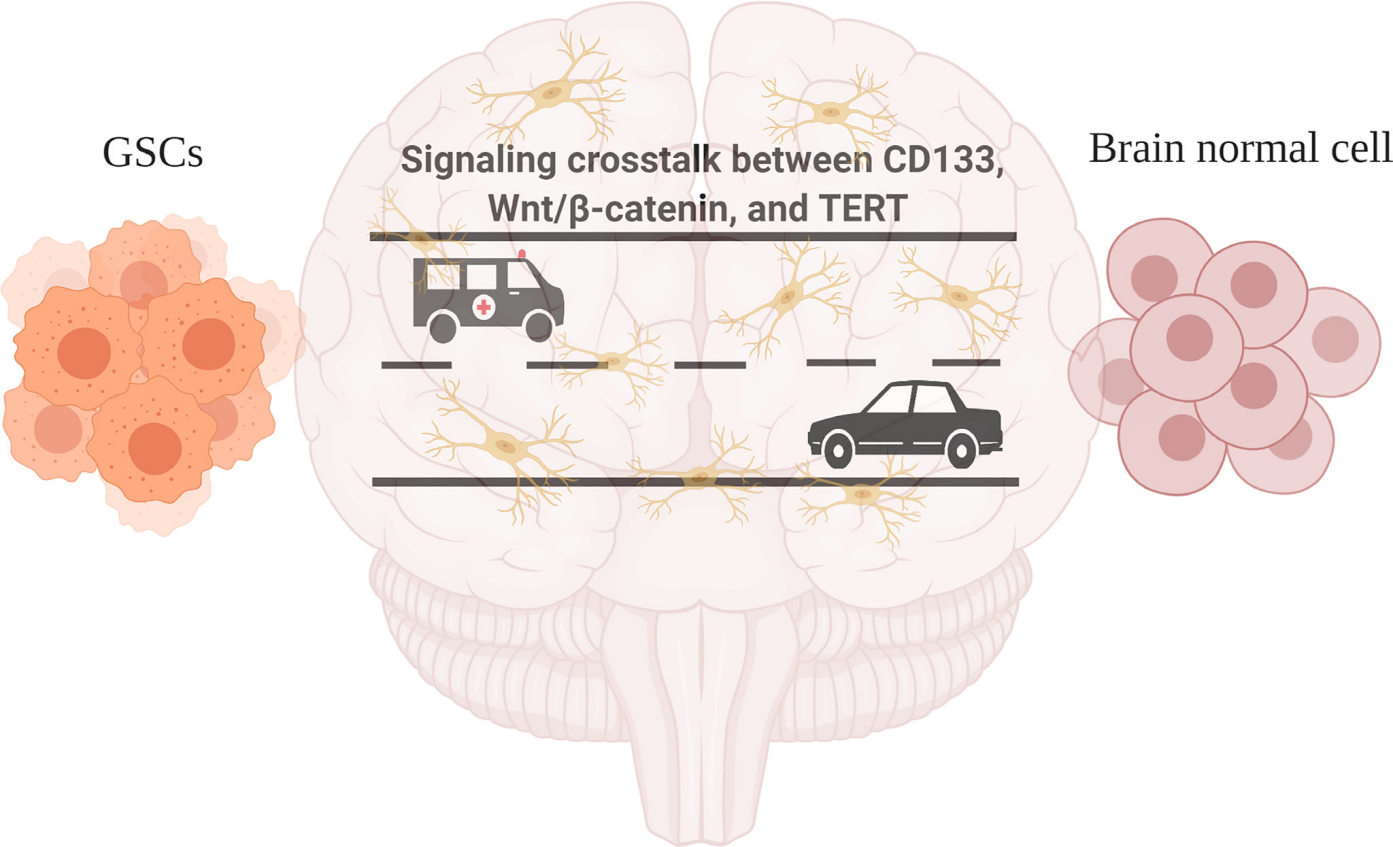
Signaling crosstalk between CD133, Wnt/β-catenin, and TERT: Rescue and Rancor in brain that is a question. (Created with BioRender.com).

The current review focuses on the significance of interplaying between CD133, Wnt/β-catenin and TERT signaling pathways in normal brain and GSCs. From a therapeutic perspective, targeting the interaction between CD133, Wnt/β-catenin and TERT will lead to the development of novel anti-cancer strategies and it would be important to examine if all these three signaling cascades can be inhibited concurrently by targeting CD133, thus killing many birds with one stone (**Figure 1**).

## General Outline of CD133 Roles in GSCs and Normal Cells

A great deal of work has been made to classify CSCs in glioblastoma using the expression of unique surface biomarkers. Human CD133 (prominin-1), a 5-transmembrance glycoprotein, is one of these biomarkers and also commonly used in identification and isolation of GSCs (13, 14). Highly expressed CD133 indicates poor outcomes among cancer patients with colorectal cancer, rectal cancer, breast cancer, lung cancer, prostate cancer, and glioblastoma (15, 16). Whilst the primary role remains uncertain, it has been presumed that CD133 could be involved in signal transduction (10). CD133 is a CSCs surface marker and has been believed to have a prognostic (17, 18) and therapeutic qualities in GSCs (15). Evidence reveals that, CD133-targeted therapy of gastric, hepatocellular carcinoma (19), and glioblastoma (20) has greatly diminished cell proliferation and tumor growth within both *in vitro* and *in vivo* CD133^POS^-marker cells. Furthermore, a study showed that CD133 knockdown could inhibit cell proliferation of glioma (21). In light of the literature review, it is evident that CD133 is imperative for the harmful oncogenic potential of GSCs as its silencing prevents both the self-renewal and tumorigenic capacities of the GBM stem cells. Notwithstanding, a shred of slightly contradictory evidence shows that some CD133^Neg^ cells can likewise develop aggressive malignancies (22). It was reported that CD133^Neg^ glioma cells give rise to tumors *in vivo* as well as CD133^Pos^ tumor cells and it was suggested that CD133 expression is needed for brain tumor initiation, however that it could be included during brain tumor progression (23). In support of this view, it was uncovered that there are CSCs in both CD133^Pos^ and CD133^Neg^ cell population originated from GBM patient, and both of CD133^Pos^ and CD133^Neg^ cells empowered the formation of tumor masses (24).

The formation of the central nervous system (CNS) of mammals, neurons, astrocytes and oligodendrocytes occurs from a reservoir of murine neuroepithelial (NE) cells, which are neural progenitor cells. Proliferative cell division changes to differentiating cell division as neurogenesis starts and it was shown that membrane particles containing CD133 have been shown to help cell differentiation. In addition to being restricted to NE progenitors, the presence of CD133 is also present in both epithelial and non-epithelial cell forms (10). Given the importance of its function it is unsurprising that it existed in most cells, however, the number of its expression is significantly different from one cell type to another. CD133 expression in normal cell is significantly lower compared to that of cancerous cells. Furthermore, this is also true when comparing normal cancer cells to their CSCs, with appear to have the highest number. CD133-mRNAs have been identified in several human tissues, like testis, digestive tract, and the pancreas, with the most notable expression in the kidney, placenta, salivary gland, and mammary gland. Non-epithelial cells, such as rod photoreceptor cells and bone marrow cells, also have CD133. CD133 appears to play a role in the formation of photoreceptor discs in this regard (10). The physiological role of CD133 in normal cell biology poorly understood. In spite of the lack of knowledge regarding the role of CD133 in normal cells, the vast majority of studies indicates that CD133 has a pivotal function in membrane organization. Moreover, the subcellular position of CD133 enables it to attach directly to the cholesterol-containing lipid rafts where it could be participated in different signaling cascade. It has been found that CD133 might be act as a scaffolding protein. Evidence indicates that, a lack of CD133 caused retinal degeneration and blindness. Additionally, it has suggested that CD133 might be has a function in maintaining of stemness properties (25). However, the exact molecular processes are still unknown. The adult mammalian brain harbors new neurons during life in two distinct locations: hippocampus and subventricular zone (SVZ). Immature neurons produced in the SVZ migrate along the rostral migratory stream (RMS) and become postmitotic interneurons in the olfactory bulb. The identity of the stem cells of the adult SVZ has been explored extensively. It was indicated that new neurons were generated by CD133^Pos^ ependymal stem cells in the adult SVZ/RMS *in vivo* and it was reported that CD133 is specifically localized for ependyma, albeit not all ependymal cells are CD133^Pos^ (26). In support of this view, the findings of another study have shown that CD133 is present neural stem cells in the embryonic brain, in the intermediate radial glial/ependymal cell type in the early postnatal stage, and in the adult brain. Based on these surveys, two competing scenarios for the origin of stem cell tumors in the brain were proposed: a derivation from CD133-expressing cells that are not usually found in the adult brain, or from CD133^Pos^ ependymal cells in the adult brain. Additionally, stem cells of brain tumors may be produced from proliferative yet CD133^Neg^ neurogenic astrocytes in adults brain (27).

## Role of Wnt Signaling Pathway in Development of GSCs and Normal Brain

**Figure 2:**
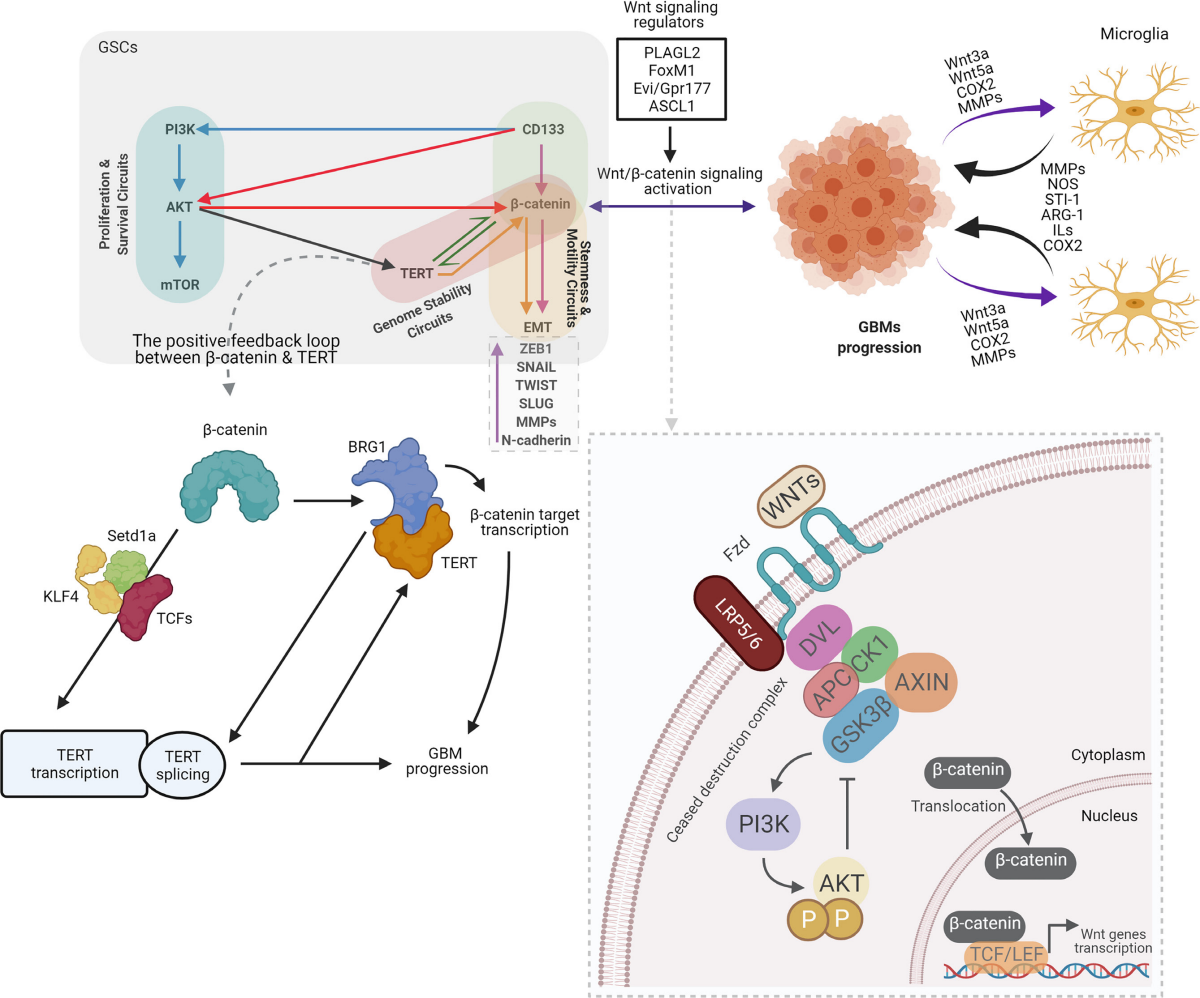
Overview of crosstalk between CD133, Wnt/β-catenin, and telomerase/TERT in GSCs, old actors and new players. By contributing to tumor growth *via* secretion of anti-inflammatory and pro-tumor factors, particularly in GBMs, Microglia benefits glioma. Several microglia-released molecules, such as STI1, epidermal growth factor (EGF), type 1 membrane matrix metalloproteinase (MT1-MMP), NOS, ARG-1, ILs, and COX2 facilitate the proliferation and migration of GBM. Meanwhile, multiple factors that recruit microglial cells and modulate their polarization are secreted by gliomas, such as Wnt3a, Wnt5a, COX2, and MMPs. GBM cells release Wnt3a in response to Wnt/β-catenin activation, which could interfere with the low-density lipoprotein receptor-related protein 5/6 (LRP5/6) and the Frizzled receptor. β-catenin stabilized and translocated to the nucleus as a result of this association, and enhanced the transcription of target genes essential for stem and cell migration (35). The Wnt/β-catenin signaling functions in GBM are defined as follows: GSCs maintenance. Wnt/β-catenin signaling regulators such as PLAGL2, FoxM1, Evi/Gpr177, and ASCL1 activate Wnt/β-catenin signaling and increase the stemness of GBM. Invasiveness of GBM cells. Wnt/β-catenin signaling activation contributes to upregulation of EMT-related genes such as ZEB1, SNAIL, TWIST, SLUG, MMPs, and N-cadherin, leading to increased migration and invasion of GBM cells (36). Several lines of evidence suggest the positive feedback loop between β-catenin and TERT. β-catenin stimulates TERT transcription directly, while TERT serves as a co-factor to facilitate the transcription of the target genes of β-catenin *via* the recruitment of BRG1, resulting in the creation of a positive feedback loop. Furthermore, BRG1 and P54 (nrb) cooperate to control TERT splicing and facilitate full-length TERT mRNA generation. It has been shown that β-catenin binds directly to the TCF site in the TERT promoter and recruit’s lysine methyltransferase Setd1a to the promoter region, while Setd1a catalyzes histone H3K4 trimethylation at the promoter site. TCF1, TCF4, and KLF4 can also be involved in β-catenin mediated TERT transcription. In the light of these findings, along with the influence of TERT on target genes for β-catenin, a positive feedback loop between TERT and β-catenin can be readily present in stem and cancer cells. However, it is not clear if BRG1 participates in the activation of TERT transcription by β-catenin, or whether TERT participates independently (12). PI3K, Phosphoinositide 3-kinases; AKT, Protein kinase B; mTOR, The mechanistic target of rapamycin; TERT, Telomerase Reverse Transcriptase; TWIST, Twist-related protein; COX2, Cyclooxygenase-2; MMPs, Matrix metalloproteinases; NOS, Nitric oxide; STI-1, Stress inducible protein-1; ILS, Interleukins; BRG1, brahma-related gene-1; setd1a, SET Domain Containing 1A; Histone Lysine Methyltransferase; KLF4, Kruppel-like factor 4; PLAGL2, Pleiomorphic adenoma gene-like 2; FoxM1, Forkhead box protein M1; Evi/GPr177, G protein-coupled receptor 177; ASCL1, Achaete-scute homolog 1; DVL, Dishevelled; APC, Adenomatous polyposis coli; CK1, The casein kinase 1; GSK3β, glycogen synthase kinase 3β. (Created with BioRender.com).

Wnt signaling pathway is mediated by Wnts proteins that are secreted by glycoproteins. This Wnts proteins are critical for cell proliferation, and differentiation of both normal and cancer stem cells (28, 29). Two kind of Wnt pathways could determine fate of the cell which are canonical Wnt pathway (Wnt/β-catenin) and non-canonical Wnt pathway. Embryonic development, cell movement, and tissue polarity are regulated by canonical and non-canonical Wnt pathways, respectively (30–32). The Wnt/β-catenin pathway is a strategically valuable molecular mechanism that provides proliferation of all types of stem cells. Increasing data show that hyperactivated-Wnt/β-catenin signaling exist in many cancers and it could adjust the self-renewal of CSCs and GSCS. It has also enhanced growth of tumor and tumor recurrence (33). As a part of the canonical Wnt pathway, β-catenin may control cell proliferation in different types of cells. Stabilized- β-catenin has been shown to translocate to the nucleus and to make T cell factor/lymphocyte enhancer factor 1 (Tcf/LEF1) complex. As a result of this complex, the target genes of Wnt, Cellular Myc (c-Myc) and Cyclin-D1, which are involved in the proliferation of glioma cells are activated (34). Intriguingly, it was speculated that the interaction of microglia-GBMs could be mediated by activated-Wnt/β-catenin pathway. Activated-Wnt/β-catenin in GBM cells might boost the recruitment of microglia by the release of Wnt3a, Wnt5a, Cyclooxygenase-2 (COX2), and metalloproteinases. As a consequence of this activation, microglia release factors such as Matrix metalloproteinases (MMPs), Nitric oxide (NOS), Stress inducible protein-1 (STI-1), Arginase-1 (ARG-1), Interleukins (ILS) and COX2 that can be influence the progression of GBM (35) (**Figure 2**).

It has been accepted that Wnt/β-catenin plays an inevitable role in neurogenesis and gliogenesis in the neocortex. Neurogenesis in neocortex occurs in the ventricular zone (VZ) of the dorsal telencephalon from radial glia (RG) and in the subventricular zone (SVZ) from neural intermediate progenitors (Ips) (37). During embryogenesis, the Wnt/β-catenin pathway is active in VZ zone (38–40). The β-catenin/TCF complex seems to specifically control the neurogenin 1 promoter, a gene involved in cortical neuronal differentiation (41). The beta-catenin expression is highest in the brain during the developmental period and the cytoplasmic level increases in the neurons at day 5 in the mice (42). The amount of beta-catenin mRNA declines in the post-developmental period, which continues in adulthood. However, beta-catenin proteins show higher levels with progressive aging (43). Regulation of beta-catenin levels during brain development is significant, as overexpression of the gene may contribute to an increase in neural growth and a decrease in differentiation, whereas premature inhibition of beta-catenin expression before its natural decline can cause the progenitor neurons to exit the cell cycle early with increased neuronal differentiation (44). Beta-catenin will act as an adhesion protein where it acts as an anchor for cadherin, which is then bound to the actin cytoskeleton of the cell by means of alpha-catenin and thereby ties the cells together (45). As an adhesion protein, beta-catenin can influence synaptic stability during the estrous period between hippocampal neurons, hypothalamic neurons, and even within the amygdala neurons, where its activation correlates with the development and consolidation of brain memory (46–48). Beta-catenin-related dysfunctions have been involved in pathological disorders such as depression, neurodegenerative diseases, and cancer (49). In addition, experimental findings have shown that the activated-Wnt/β-catenin pathway increases the formation of tight junction proteins in the developing brain capillaries (50–52). However, the precise function of Wnt/β-catenin in GSCs and normal brain cells remains poorly elucidated.

## Functions of Telomerase/TERT in GSCs and Normal Brain

It subsists ambiguous what self-defense mechanism are applied by GSCs against chemotherapeutic drugs. As of lately, it is believed that the DNA damage response (DDR) and DNA repair pathway have undeniable role in self-defense mechanism of cancer stem cells (CSCs) (53). Growing evidence demonstrated that direct correlation exists between elevated DDR and chemoresistance of the CSCs. In light of new observations indicating that maintaining genome stability CSCs might be on account of activation of DDR pathways. Activation of telomerase is one of the critical DDR pathways in GSCs (54). Guanine-rich repeated sequences are attached to the chromosomal terminals using the RNA template by telomerase which recompense the loss of DNA replication. Slight or no activity were reported for telomerase in normal human somatic cells (55). The enhanced activated-telomerase enzyme is one of the most significant molecular properties of cancer. Reactivated telomerase was shown in approximately 90% of tumors (56). It composed of catalytic protein telomerase reverse transcriptase (TERT) and RNA template (TERC). Irrespective of telomerase function, the TERC subunit is expressed in most cell types and the TERT subunit is heavily regulated through cell differentiation. The expression of TERT and TERC in normal cells is small, but the expression of TERT and TERC in tumor cells is indeed very substantial (54, 57). Telomerase is a reverse transcriptase enzyme that has a canonical and non-canonical function in cancer cells. The best-known canonical function of telomerase is maintaining telomere, and the non-canonical functions include DNA repair, anti-apoptotic activity, protection of mitochondrial DNA against oxidative stress, and pro-proliferative effects (58, 59).

In the majority of human primary cancers (~90%), TERT expression/telomerase activity is perceptible. Additionally, induced-TERT or activated-telomerase bestows immortality properties to cancer cells by stabilizing their telomere length (TL). However, current surveys have indicated that its oncogenic features independently of TL, which include DNA damage repair, gene transcription and microRNA expression (60). Moreover, mutated-TERT promoter have been observed in more than 50% of primary adult GBMs. Reports have pointed out that TERT was able to enhance stemness of glioma cells by modulation of epidermal growth factor receptor (EGFR) expression. Inversely, down-regulated TERT expression was coincided with declined expression of EGFR, basic fibroblast growth factor (bFGF) and glioma stem cell properties (61). How the molecular mechanistic details of TERT be able to modulate stemness of glioma remains unclear. Some suggested that GSCs have common properties with astrocyte-like neural stem cells (NSCs) of the SVZ and GBM might spring from the somatic mutations in NSCs of the SVZ. Thus, mutated-TERT promoter in NSCs could license them to expand a self-renewal capacity (62, 63).

Does activated-telomerase and expressed-TERT guard the neurons in our brain? Activated-telomerase is conjoined with cell proliferation during mammalian embryonic development, cell transformation, and in cancers. In adult animals the expression of telomerase is limited to the subventricular zone (SVZ) and olfactory bulb (62). It was shown that the proliferative capacity of somatic cells could be boosted by the up-regulation of TERT. Moreover, as somatic stem cells differentiate, the activity of telomerase and the expression of TERT decline (64, 65). The neurogenesis investigations have indicated that the activity of telomerase and expression of TERT are incremented in neuronal precursor cells in rats and mice brains during embryonic development. Furthermore, the declined-telomerase activity has coincided with a reduction of proliferation of neuroblasts, but the expression of TERT maintains boosted for an extended time period as neurons differentiate and migrate (66). Intriguingly, it was demonstrated that the activity of telomerase is increased in responses to brain injury and it seems that telomerase activity is enhanced in microglia and stem cells because brain tissue injury can induce proliferation of these cells (67).

## The Signaling Crosstalk Between CD133, Wnt/β-Catenin, and TERT in Normal Brain

Meanwhile, CD133, also known as prominin-1, is one of the most used biomarkers for the isolation of cancer stem cells (CSCs). The rigorous function of CD133 maintains unidentified, but it has been proposed that it would perform as a cell membrane topology organizer. Furthermore, by activating the Wnt/β-catenin pathway, CD133 could enhance growth, differentiation of nerve cells, and neurogenesis in the brain (10). The activated-Wnt/β-catenin pathway modulates brain development and adult function such as regulation of dendrite formation and synaptic function. By considering the findings stated above, the activated CD133-Wnt/β-catenin has a critical role in the function and integrity of adult brain (68). Importantly, it was reported that TERT has an ability to interact with brahma-related gene-1 (BRG1), chromatin-remodeling factor, and facilitates recruiting BRG1 to β-catenin genes for their transcriptional activation. Thus, the activated-TERT-Wnt/β-catenin axis might increase the proliferation of normal mouse stem cells (60, 69).

## The Signaling Crosstalk Between CD133, Wnt/β-Catenin, and TERT Might Drive GSCs

Up-regulated CD133 in GBM, has been linked to the self-renewal ability of CSCs and chemotherapy resistance. As well as, it has demonstrated that overexpressed-CD133 is associated with GBM progression and tumorigenesis (70). The findings of study showed that the Wnt/β-catenin pathway could control the activity of GSCs. In this study, the expression levels of Wnt/β-catenin pathway proteins in CD133^Pos^CSC and CD133^Neg^ differentiated glioblastoma cells (DGCs) were compared (*P ≤ 0.05*). The expression of eight Wnt proteins (APC, CSNK1E, CSNK1A, CSNK2A2, CSNK2B, CTNNB1, DVL1, RUVBL) was substantially increased. Interestingly, the expression of CTNNB1 (β-catenin) was enhanced 13.98-fold in CD133^Pos^CSC relative to CD133^Neg^ DGCs (71). Data suggest that CD133 is one of the main regulators for β-catenin signaling. Recently, CD133 has been shown to improve the proliferation of clones and the repair of kidneys by regulating the Wnt pathway *via* the modulation of β-catenin levels (72). Another research in liver cancers found that the risen clonogenic ability of CSCs is associated with modulation in Wnt/β-catenin signaling, which is positively linked to CD133 expression. It has been indicated that the CD133-Wnt/β-catenin axis has a critical role in regulating CSCs (73).

Interestingly, the Wnt/β-catenin pathway can do upregulate the stemness of brain cancer by modulating the CD44 CSC marker. It is important to note that Wnt/β-catenin highly activated sub-population of GSCs showed enhanced levels of expression of genes CD133, SRY-box 2 (SOX2), Nanog, Octamer-binding transcription factor-4 (Oct-4), and Nestin, which proposes the role of biomarkers-Wnt/β-catenin axis in remaining stemness traits of GBM (74). Additionally, the results of the study have indicated that CD133 may conceivably activate Wnt/β-catenin signaling pathway through AKT and leads to promote brain tumor-initiating cells in GBM. In other words, the outcome has shown that the CD133-AKT- Wnt/β-catenin axis drives glioblastoma-initiating cell tumor (4). The level of phosphorylate-Akt in CD133^Pos^ cancer cells is greater than in CD133^Neg^ cancer cells, notably in GSCs (5). Consequently, based on this, we may assume CD133 to be an activator of the PI3K-Akt pathway. The 5’ regulatory area of TERT contains multiple binding sites for transcription factors, such as Wnt/β-catenin, c-Myc, estrogen receptor, Activator protein-1 (AP1), Signal transducer and activator of transcription proteins (STAT), Mas1 and Pax (Paired Box Proteins) (75–77). The PI3K/Akt/mTOR pathway enhances the expression of TERT thru many pathways (78). Activated-AKT through preventing interactions between mouse double minute 2 homolog (MDM2) and p14 (p19), induces a cell cycle that could impede ubiquitin-mediated p53 proteolysis and the mTOR-mediated degradation of the c-Myc competitor mitotic arrest deficiency-1 (MAD1) (79). Some investigations have shown that overexpressed-c-Myc oncogene plays a key role in the induction of telomerase enzyme activity by increasing TERT expression (80, 81).

Growing data has shown that TERT has mechanistically occupied Wnt/β-catenin promoters, including cyclin D1 and c-Myc. Of note, c-Myc (TERT stimulator) may be regulated by Wnt/β-catenin. On one hand, TERT may be regarded as one of the key targets of the Wnt/β-catenin signaling pathway. It has been shown that β-catenin can interact directly with the mouse and human cancer cells or cancer stem cells TERT promoter. In plain terms, these findings indicate that β-catenin controls the length of telomere by activating the transcription of TERT. Another research stated that TERT could stimulate EMT using Wnt/β-catenin. In addition, *in vitro* research findings showed that TERT expression levels were associated with Snail1 and vimentin expression levels (82–86). TERT activation is an essential step in GBM tumorigenesis and it was shown that TERT permits GBMs to attain CSC characteristics by inducing EGFR expression (61, 87). Of note, the investigations have revealed that TERT has direct interaction with β-catenin and presumably enhance transcriptional outputs of it. Besides, activated-TERT- β-catenin axis mayhap stimulate epithelial-mesenchymal transformation (EMT) in CSCs (54, 86, 88). CSCs and TERT could be linked together by Wnt/β-catenin pathway (85, 89). In summary, the crosstalk between each component of the CD133-Wnt/β-catenin-TERT axis may be considered a pivotal regulatory signaling pathway in the CSCs and GSCs.

## Conclusion

In a nutshell, in the normal brain, each element of signaling pathways of CD133, Wnt/β-catenin, and TERT has a critical role in the integrity of the brain It is generally accepted that the expression of CD133 in normal cell is significantly lower compared to cancerous cells. This is also true and when comparing normal cancer cells to GSCs (i.e., expression in normal cancer cells is significantly less), especially in GSCs which has the highest expression. Upon minimizing toxicity effect towards normal cells targeting therapy is important. One can target CD133 surface receptor to specifically bring drugs to CSCs leveraging on its significantly high expression in CSCs. The overexpressed-TERT would increase cell proliferation during embryonic development, whilst CD133 biomarker by activating Wnt/β-catenin pathway leads to neurogenesis. Thus, might be the cross-talk between each component of the mentioned axis has a neuroprotective effect on the normal brain. On the one hand, CD133^pos/high^ sub-population of GSCs correlates to invasiveness and progression of tumor and besides, CD133-Wnt/β-catenin axis has an inevitable role in stemness of GBM. Recent evidence also demonstrated that interplay between Wnt/β-catenin pathway and TERT can be modulating the stemness of CSCs. Hence, activation of the above axis in the brain might be considered as a gate between “tranquility and turmoil” and targeting this pathway might shed a light on GBM therapy and prompt further investigations in this area. However, the bulk of these targeting techniques are of an experimental nature and their medical use is minimal. The biggest challenge is the heterogeneity of GSC’s surface biomarkers, which render it challenging to detect and therefore aim therapy. In addition, answering the challenge of similarities between normal neural stem cells and GSCs makes treatment-associated toxicity possible.

## Author Contributions

AB: Conceptualization, Writing – Original draft. AS: Supervision, Writing – Reviewing, and Editing, Funding Acquisition. All authors contributed to the article and approved the submitted version.

## Conflict of Interest

The authors declare that the research was conducted in the absence of any commercial or financial relationships that could be construed as a potential conflict of interest.

## References

[1] Bhaduri A Di Lullo E Jung D Müller S Crouch EE Espinosa CS. Outer Radial Glia-like Cancer Stem Cells Contribute to Heterogeneity of Glioblastoma. Cell Stem Cell (2020) 26:48–63. 10.1016/j.stem.2019.11.015 31901251 PMC7029801

[2] Gimple RC Bhargava S Dixit D Rich JN. Glioblastoma stem cells: Lessons from the tumor hierarchy in a lethal cancer. Genes Dev (2019) 33:591–609. 10.1101/gad.324301.119 31160393 PMC6546059

[3] Auffinger B Spencer D Pytel P Ahmed AU Lesniak MS. The role of glioma stem cells in chemotherapy resistance and glioblastoma multiforme recurrence. Expert Rev Neurotherapeut (2015) 15:741–52. 10.1586/14737175.2015.1051968 PMC4830899 26027432

[4] Manoranjan B Chokshi C Venugopal C Subapanditha M Savage N Tatari N. A CD133-AKT-Wnt signaling axis drives glioblastoma brain tumor-initiating cells. Oncogene (2020) 39:1590–99. 10.1038/s41388-019-1086-x 31695152

[5] Wei Y Jiang Y Liu Y Zou F Liu YC Wang S. Activation of PI3K/Akt pathway by CD133-p85 interaction promotes tumorigenic capacity of glioma stem cells. Proc Natl Acad Sci USA (2013) 110:6829–34. 10.1073/pnas.1217002110 PMC3637720 23569237

[6] Aghajani M Mansoori B Mohammadi A Asadzadeh Z Baradaran B. “New emerging roles of CD133 in cancer stem cell: Signaling pathway and miRNA regulation,”. J Cell Physiol (2019) 234:21642–661. 10.1002/jcp.28824 31102292

[7] Mak AB Nixon AML Kittanakom S Stewart JM Chen GI Curak J. Regulation of CD133 by HDAC6 Promotes β-Catenin Signaling to Suppress Cancer Cell Differentiation. Cell Rep (2012) 2:951–63. 10.1016/j.celrep.2012.09.016 PMC3590846 23084749

[8] Sastre-Perona A Riesco-Eizaguirre G Zaballos MA Santisteban P. β-catenin signaling is required for RAS-driven thyroid cancer through PI3K activation. Oncotarget (2016) 7:49435–449. 10.18632/oncotarget.10356 PMC5226519 27384483

[9] Ghareghomi S Ahmadian S Zarghami N. Biochimie Fundamental insights into the interaction between telomerase/TERT and intracellular signaling pathways. Biochimie (2021) 181:12–24. 10.1016/j.biochi.2020.11.015 33232793

[10] Barzegar Behrooz A Syahir A Ahmad S. CD133: beyond a cancer stem cell biomarker. J Drug Targeting (2018) 27:257–69. 10.1080/1061186X.2018.1479756 29911902

[11] Chenn A. Wnt/β-catenin signaling in cerebral cortical development. Organogenesis (2008) 4:76–80. 10.4161/org.4.2.5852 19279718 PMC2634251

[12] Yuan X Xu D. Telomerase reverse transcriptase (TERT) in action: Cross-talking with epigenetics. Int J Mol Sci (2019) 20:3338. 10.3390/ijms20133338 PMC6651578 31284662

[13] Singh SK Clarke ID Terasaki M Bonn VE Hawkins C Squire J. Identification of a cancer stem cell in human brain tumors. Cancer Res (2003) 63:5821–28. 14522905

[14] Singh SK Hawkins C Clarke ID Squire JA Bayani J Hide T. Identification of human brain tumour initiating cells. Nature (2004) 432:396–401. 10.1038/nature03128 15549107

[15] Liou GY. CD133 as a regulator of cancer metastasis through the cancer stem cells. Int J Biochem Cell Biol (2019) 106:1–7. 10.1016/j.biocel.2018.10.013 30399449 PMC6309463

[16] Colman H Zhang L Sulman EP McDonald M Shooshtari NL Rivera A. A multigene predictor of outcome in glioblastoma. Neuro Oncol (2010) 12:49–57. 10.1093/neuonc/nop007 20150367 PMC2940562

[17] Li B McCrudden CM Yuen HF Xi X Lyu P Chan KW. CD133 in brain tumor: The prognostic factor. Oncotarget (2017) 11144–59. 10.18632/oncotarget.14406 PMC5355253 28055976

[18] Pallini R Ricci-Vitiani L Montano N Mollinari C Biffoni M Cenci T. Expression of the stem cell marker CD133 in recurrent glioblastoma and its value for prognosis. Cancer (2011) 117:162–174. 10.1002/cncr.25581 20806346

[19] Smith LM Nesterova A Ryan MC Duniho S Jonas M Anderson M. CD133/prominin-1 is a potential therapeutic target for antibody-drug conjugates in hepatocellular and gastric cancers. Br J Cancer (2008) 99:100–9. 10.1038/sj.bjc.6604437 PMC2453027 18542072

[20] Vora P Venugopal C Salim SK Tatari N Bakhshinyan D Singh M. The Rational Development of CD133-Targeting Immunotherapies for Glioblastoma. Cell Stem Cell (2020) 26:832–44. 10.1016/j.stem.2020.04.008 32464096

[21] Yao J Zhang T Ren J Yu M Wu G. Effect of CD133/prominin-1 antisense oligodeoxynucleotide on *in vitro* growth characteristics of Huh-7 human hepatocarcinoma cells and U251 human glioma cells. Oncol Rep (2009) 22:781–87. 10.3892/or_00000500 19724856

[22] Ahmed SI Javed G Laghari AA Bareeqa SB Farrukh S Zahid S. CD133 Expression in Glioblastoma Multiforme: A Literature Review. Cureus (2018) 10:e3439. 10.7759/cureus.3439 30555755 PMC6290980

[23] Wang J Sakariassen P Tsinkalovsky O Immervoll H Bøe SO Svendsen A. CD133 negative glioma cells form tumors in nude rats and give rise to CD133 positive cells. Int J Cancer (2008) 122:761–68. 10.1002/ijc.23130 17955491

[24] Joo KM Kim SY Jin X Song SY Kong DS Lee JI. Clinical and biological implications of CD133-positive and CD133-negative cells in glioblastomas. Lab Investig (2008) 88:808–15. 10.1038/labinvest.2008.57 18560366

[25] Glumac PM LeBeau AM. The role of CD133 in cancer: a concise review. Clin Transl Med (2018) 7:18. 10.1186/s40169-018-0198-1 PMC6035906 29984391

[26] Coskun V Wu H Blanchi B Tsao S Kim K Zhao J. CD133+ neural stem cells in the ependyma of mammalian postnatal forebrain. Proc Natl Acad Sci U S A (2008) 105:1026–1031. 10.1073/pnas.0710000105 18195354 PMC2242680

[27] Pfenninger CV Roschupkina T Hertwig F Kottwitz D Englund E Bengzon J. CD133 is not present on neurogenic astrocytes in the adult subventricular zone, but on embryonic neural stem cells, ependymal cells and glioblastoma cells. Cancer Res (2007) 67:5727–36. 10.1158/0008-5472.CAN-07-0183 17575139

[28] Anastas JN Moon RT. WNT signalling pathways as therapeutic targets in cancer. Nat Rev Cancer (2013) 13:11–26. 10.1038/nrc3419 23258168

[29] Reya T Clevers H. Wnt signalling in stem cells and cancer. Nature (2005) 434:843–50. 10.1038/nature03319 15829953

[30] Zhang L Yang X Yang S Zhang J. The Wnt/β-catenin signaling pathway in the adult neurogenesis. Eur J Neurosci (2011) 33:1–8. 10.1111/j.1460-9568.2010.7483.x 21073552

[31] Nam JS Turcotte TJ Smith PF Choi S Jeong KY. Mouse cristin/R-spondin family proteins are novel ligands for the frizzled 8 and LRP6 receptors and activate β-catenin-dependent gene expression. J Biol Chem (2006) 281:13247–57. 10.1074/jbc.M508324200 16543246

[32] Guan R Zhang X Guo M. “Glioblastoma stem cells and Wnt signaling pathway: Molecular mechanisms and therapeutic targets. Chin Neurosurg J (2020) 6:25. 10.1186/s41016-020-00207-z 32922954 PMC7398200

[33] Mao J Fan S Ma W Fan P Wang B Zhang J. Roles of Wnt/β-catenin signaling in the gastric cancer stem cells proliferation and salinomycin treatment. Cell Death Dis (2014) 5:e1039. 10.1038/cddis.2013.515 24481453 PMC4040703

[34] Nager M Bhardwaj D Cantí C Medina L Nogués P Herreros J. β -Catenin Signalling in Glioblastoma Multiforme and Glioma-Initiating Cells. Chemother Res Pract (2012) 2012:7. 10.1155/2012/192362 PMC3286890 22400111

[35] Matias D Predes D Niemeyer Filho P Lopes MC Abreu JG Lima FRS. Microglia-glioblastoma interactions: New role for Wnt signaling. Biochim Biophys Acta Rev Cancer (2017) 1868:333–40. 10.1016/j.bbcan.2017.05.007 28554667

[36] Lee Y Lee JK Ahn SH Lee J Nam DH. WNT signaling in glioblastoma and therapeutic opportunities. Lab Investig (2016) 96:137–50. 10.1038/labinvest.2015.140 26641068

[37] Bem J Brożko N Chakraborty C Lipiec MA Koziński K Nagalski A. Wnt/β-catenin signaling in brain development and mental disorders: keeping TCF7L2 in mind. FEBS Lett (2019) 593:1654–74. 10.1002/1873-3468.13502 PMC6772062 31218672

[38] Grove EA Tole S Limon J Yip LW Ragsdale CW. The hem of the embryonic cerebral cortex is defined by the expression of multiple Wnt genes and is compromised in Gli3-deficient mice. Development (1998) 125:2315–25. 10.1242/dev.125.12.2315 9584130

[39] Chodelkova O Masek J Korinek V Kozmik Z Machon O. Tcf7L2 is essential for neurogenesis in the developing mouse neocortex. Neural Dev (2018) 13:8. 10.1186/s13064-018-0107-8 29751817 PMC5946422

[40] Machon O Van Den Bout CJ Backman M Kemler R Krauss S. Role of β-catenin in the developing cortical and hippocampal neuroepithelium. Neuroscience (2003) 122:129–43. 10.1016/S0306-4522(03)00519-0 14596855

[41] Hirabayashi Y Itoh Y Tabata H Nakajima K Akiyama T Masuyama N. The Wnt/β-catenin pathway directs neuronal differentation of cortical neural precursor cells. Development (2004) 131:2791–801. 10.1242/dev.01165 15142975

[42] Coyle-Rink J Del Valle L Sweet T Khalili K Amini S. Developmental expression of Wnt signaling factors in mouse brain. Cancer Biol Ther (2002) 1:640–45. 10.4161/cbt.313 12642687

[43] Lu T Aron L Zullo J Pan Y Kim H Chen Y. REST and stress resistance in ageing and Alzheimer’s disease. Nature (2014) 507:448–54. 10.1038/nature13163 PMC4110979 24670762

[44] Chenn A Walsh CA. Regulation of cerebral cortical size by control of cell cycle exit in neural precursors. Science (2002) 297:365–69. 10.1126/science.1074192 12130776

[45] Drees F Pokutta S Yamada S Nelson WJ Weis WI. α-catenin is a molecular switch that binds E-cadherin-β-catenin and regulates actin-filament assembly. Cell (2005) 123:903–15. 10.1016/j.cell.2005.09.021 PMC3369825 16325583

[46] Maguschak KA Ressler KJ. β-catenin is required for memory consolidation. Nat Neurosci (2008) 11:1319–26. 10.1038/nn.2198 PMC2597638 18820693

[47] Mills F Bartlett TE Dissing-Olesen L Wisniewska MB Kuznicki J Macvicar BA. Cognitive flexibility and long-term depression (LTD) are impaired following β-catenin stabilization *in vivo* . Proc Natl Acad Sci USA (2014) 111:8631–36. 10.1073/pnas.1404670111 PMC4060709 24912177

[48] Barrera-Ocampo A Gutierrez-Vargas J Garcia-Segura LM Cardona-Gómez GP. Glycogen synthase kinase-3β/β-catenin signaling in the rat hypothalamus during the estrous cycle. J Neurosci Res (2012) 90:1078–84. 10.1002/jnr.22816 22331547

[49] Teo CH Soga T Parhar IS. Brain Beta-Catenin Signalling during Stress and Depression. NeuroSignals (2019) 26:31–42. 10.1159/000487764 29490303

[50] Daneman R Agalliu D Zhou L Kuhnert F Kuo CJ Barres BA. Wnt/β-catenin signaling is required for CNS, but not non-CNS, angiogenesis. Proc Natl Acad Sci USA (2009) 106:641. 10.1073/pnas.0805165106 PMC2626756 19129494

[51] Harati R Benech H Villégier AS Mabondzo A. P-glycoprotein, breast cancer resistance protein, organic anion transporter 3, and transporting peptide 1a4 during blood-brain barrier maturation: Involvement of Wnt/β-catenin and endothelin-1 signaling. Mol Pharm (2013) 10:1566–80. 10.1021/mp300334r 22998451

[52] Laksitorini MD Yathindranath V Xiong W Hombach-Klonisch S Miller DW. Modulation of Wnt/β-catenin signaling promotes blood-brain barrier phenotype in cultured brain endothelial cells. Sci Rep (2019) 9:19718. 10.1038/s41598-019-56075-w 31873116 PMC6928218

[53] Abad E Graifer D Lyakhovich A. DNA damage response and resistance of cancer stem cells. Cancer Lett (2020) 1:106–17. 10.1016/j.canlet.2020.01.008 31968219

[54] Liu N Guo XH Liu JP Cong YS. Role of telomerase in the tumour microenvironment. Clin Exp Pharmacol Physiol (2020) 47:357–64. 10.1111/1440-1681.13223 31799699

[55] Parkinson EK. Telomerase as a novel and potentially selective target for cancer chemotherapy. Ann Med (2003) 35:466–75. 10.1080/07853890310006361 14649329

[56] Podlevsky JD Bley CJ Omana RV Qi X Chen JL. The Telomerase Database. Nucleic Acids Res (2008) 36:D339–43. 10.1093/nar/gkm700 PMC2238860 18073191

[57] Jiang J Chan H Cash DD Miracco EJ Ogorzalek Loo RR Upton HE. Structure of Tetrahymena telomerase reveals previously unknown subunits, functions, and interactions. Science (2015) 350:aab4070. 10.1126/science.aab4070 26472759 PMC4687456

[58] Saretzki G. Does telomerase protein protect our neurons? J Neurol Neuromed (2016) 1:23–8. 10.29245/2572.942X/2016/2.1025

[59] Lu C Fu W Mattson MP. Telomerase protects developing neurons against DNA damage-induced cell death. Dev Brain Res (2001) 131:167–71. 10.1016/S0165-3806(01)00237-1 11718848

[60] Yuan X Larsson C Xu D. Mechanisms underlying the activation of TERT transcription and telomerase activity in human cancer: old actors and new players. Oncogene (2019) 38:6172–83. 10.1038/s41388-019-0872-9 PMC6756069 31285550

[61] Beck S Jin X Sohn YW Kim JK Kim SH Yin J. Telomerase activity-independent function of TERT allows glioma cells to attain cancer stem cell characteristics by inducing EGFR expression. Mol Cells (2011) 31:9–15. 10.1007/s10059-011-0008-8 21193962 PMC3906874

[62] Matarredona ER Pastor AM. Neural stem cells of the subventricular zone as the origin of human glioblastoma stem cells. Therapeutic implications. Front Oncol (2019) 9:799. 10.3389/fonc.2019.00779 31482066 PMC6710355

[63] Sanai N Alvarez-Buylla A Berger MS. Neural Stem Cells and the Origin of Gliomas. N Engl J Med (2005) 353:811–22. 10.1056/NEJMra043666 16120861

[64] Greenberg RA Allsopp RC Chin L Morin GB DePinho RA. Expression of mouse telomerase reverse transcriptase during development, differentiation and proliferation. Oncogene (1998) 16:1723–30. 10.1038/sj.onc.1201933 9582020

[65] Bodnar AG Ouellette M Frolkis M Holt SE Chiu CP Morin GB. Extension of life-span by introduction of telomerase into normal human cells. Science (1998) 279:349–52. 10.1126/science.279.5349.349 9454332

[66] Klapper W Shin T Mattson MP. Differential regulation of telomerase activity and TERT expression during brain development in mice. J Neurosci Res (2001) 64:252–60. 10.1002/jnr.1073 11319769

[67] Liu J Solway K Messing RO Sharp FR. Increased neurogenesis in the dentate gyrus after transient global ischemia in gerbils. J Neurosci (1998) 18:7768–78. 10.1523/JNEUROSCI.18-19-07768.1998 PMC6793017 9742147

[68] Zuccarini M Giuliani P Ziberi S Carluccio M Di Iorio P Caciagli F. The role of wnt signal in glioblastoma development and progression: A possible new pharmacological target for the therapy of this tumor. Genes (2018) 9:105. 10.3390/genes9020105 PMC5852601 29462960

[69] Il Park J Venteicher AS Hong JY Choi J Jun S Shkreli M. Telomerase modulates Wnt signalling by association with target gene chromatin. Nature (2009) 460:66–72. 10.1038/nature08137 19571879 PMC4349391

[70] Hassn Mesrati M Behrooz AB Abuhamad AY Syahir A. Understanding Glioblastoma Biomarkers: Knocking a Mountain with a Hammer. Cells (2020) 9:1236. 10.3390/cells9051236 PMC7291262 32429463

[71] Shevchenko V Arnotskaya N Zaitsev S Sharma A Sharma HS Bryukhovetskiy A. Proteins of Wnt signaling pathway in cancer stem cells of human glioblastoma. In: International Review of Neurobiology, vol. 151. (2020). p. 185–200. 10.1016/bs.irn.2020.03.006 32448607

[72] Brossa A Papadimitriou E Collino F Incarnato D Oliviero S Camussi G. Role of CD133 Molecule in Wnt Response and Renal Repair. Stem Cells Transl Med (2018) 7:283–94. 10.1002/sctm.17-0158 PMC5827750 29431914

[73] Aghajani M Mansoori B Mohammadi A Asadzadeh Z Baradaran B. New emerging roles of CD133 in cancer stem cell: Signaling pathway and miRNA regulation. J Cell Physiol (2019) 234:21642–661. 10.1002/jcp.28824 31102292

[74] Prasad S Ramachandran S Gupta N Kaushik I Srivastava SK. Cancer cells stemness: A doorstep to targeted therapy. Biochim Biophys Acta Mol Basis Dis (2020) 18866:165424. 10.1016/j.bbadis.2019.02.019 30818002

[75] Artandi SE Alson S Tietze MK Sharpless NE Ye S Greenberg RA. Constitutive telomerase expression promotes mammary carcinomas in aging mice. Proc Natl Acad Sci U S A (2002) 99:8191–96. 10.1073/pnas.112515399 PMC123043 12034875

[76] González-Suárez E Samper E Ramírez A Flores JM Martín-Caballero J Jorcano JL. Increased epidermal tumors and increased skin wound healing in transgenic mice overexpressing the catalytic subunit of telomerase, mTERT, in basal keratinocytes. EMBO J (2001) 20:2619–2630. 10.1093/emboj/20.11.2619 11387197 PMC125492

[77] Ramlee MK Wang J Toh WX Li S. Transcription regulation of the human telomerase reverse transcriptase (hTERT) gene. Genes (2016) 7:50. 10.3390/genes7080050 PMC4999838 27548225

[78] Daniel M Peek GW Tollefsbol TO. Regulation of the human catalytic subunit of telomerase (hTERT). Gene (2012) 498:135–46. 10.1016/j.gene.2012.01.095 PMC3312932 22381618

[79] Peek GW Tollefsbol TO. Down-regulation of hTERT and Cyclin D1 transcription *via* PI3K/Akt and TGF-β pathways in MCF-7 Cancer cells with PX-866 and Raloxifene. Exp Cell Res (2016) 344:95–102. 10.1016/j.yexcr.2016.03.022 27017931 PMC4879042

[80] Wu KJ Grandori C Amacker M Simon-Vermot N Polack A Lingner J. Direct activation of TERT transcription by c-MYC. Nat Genet (1999) 21:220–4. 10.1038/6010 9988278

[81] Khattar E Tergaonkar V. Transcriptional regulation of telomerase reverse transcriptase (TERT) by MYC. Front Cell Dev Biol (2017) 5:1. 10.3389/fcell.2017.00001 28184371 PMC5266692

[82] Li J Huang X Xie X Wang J Duan M. Human telomerase reverse transcriptase regulates cyclin D1 and G1/S phase transition in laryngeal squamous carcinoma. Acta Otolaryngol (2011) 131:546–51. 10.3109/00016489.2011.557393 21492065

[83] Yang C Przyborski S Cooke MJ Zhang X Stewart R Anyfantis G. A Key Role for Telomerase Reverse Transcriptase Unit in Modulating Human Embryonic Stem Cell Proliferation, Cell Cycle Dynamics, and In Vitro Differentiation. Stem Cells (2008) 26:850–63. 10.1634/stemcells.2007-0677 18203676

[84] Zhang Y Toh LL Lau P Wang X. Human telomerase reverse transcriptase (hTERT) is a novel target of the Wnt/β-catenin pathway in human cancer. J Biol Chem (2012) 287:32494–511. 10.1074/jbc.M112.368282 PMC3463325 22854964

[85] Hoffmeyer K Raggioli A Rudloff S Anton R Hierholzer A Del Valle I. Wnt/β-catenin signaling regulates telomerase in stem cells and cancer cells. Science (2012) 336:1549–54. 10.1126/science.1218370 22723415

[86] Liu Z Li Q Li K Chen L Li W Hou M. Telomerase reverse transcriptase promotes epithelial-mesenchymal transition and stem cell-like traits in cancer cells. Oncogene (2013) 32:4203–13. 10.1038/onc.2012.441 23045275

[87] Pestana A Vinagre J Sobrinho-Simões M Soares P. TERT biology and function in cancer: Beyond immortalisation. J Mol Endocrinol (2017) 58:R129–46. 10.1530/JME-16-0195 28057768

[88] Zhang K Guo Y Wang X Zhao H Ji J Cheng C. WNT/β-catenin directs self-renewal symmetric cell division of hTERThigh prostate cancer stem cells. Cancer Res (2017) 77:2534–47. 10.1158/0008-5472.CAN-16-1887 28209613

[89] Greider CW. Wnt regulates TERT - Putting the horse before the cart. Science (2012) 336:1519–20. 10.1126/science.1223785 22723405

